# Persistent organic pollutants and haematological markers in Greenlandic pregnant women: the ACCEPT sub-study

**DOI:** 10.1080/22423982.2018.1456303

**Published:** 2018-03-29

**Authors:** Ane-Kersti Skaarup Knudsen, Manhai Long, Henning Sloth Pedersen, Eva Cecilie Bonefeld-Jørgensen

**Affiliations:** a Centre for Arctic Health & Molecular Epidemiology, Department of Public Health, Aarhus University, Aarhus, Denmark; b Emergency Department, Regional Hospital of Randers, Randers, Denmark; c Primary Health Care Center, Queen Ingrid Hospital, Nuuk, Greenland; d Greenland Center for Health Research, University of Greenland, Nuuk, Greenland

**Keywords:** Persistent organic pollutants, blood samples, haematological markers, Inuit, pregnancy

## Abstract

The Arctic populations have high blood concentrations of persistent organic pollutants (POPs). Exposure to POPs was related to adverse health effects e.g. immune, neurological and reproductive systems. This study investigates associations between serum POP levels and haematological markers in Greenlandic pregnant women. This cross-sectional study included 189 women enrolled in 2010–2011 at the Greenlandic West coast by the inclusion criteria ≥18 years of age and had lived for 50% or more of their life in Greenland. The associations between the sum of the POP variables polychlorinated biphenyls (sumPCBs), organochlorine pesticides (sumOCPs), perfluoroalkylated substances (sumPFASs) and 24 haematological markers were analysed using linear regression adjusted for age, pre-pregnancy BMI, parity, gestation week, plasma-cotinine and alcohol intake. It showed a significantly inverse association between several haematological markers (eosinophil, lymphocyte, neutrophil and white blood cells) and sumPCBs, sumOCPs and sumPFASs. In addition, the monocyte, mean corpuscular haemoglobin concentration, plateletcrit and platelet count markers were significantly inversely associated with sumPFASs, but the haematocrit and mean erythrocyte corpuscular volume were positively associated with sumPFASs. In conclusion, exposure to POPs influenced several haematological markers, especially cell count parameters, suggesting immunosuppressive potential of POPs in Greenlandic pregnant women. The data need further investigations.

## Introduction

The environmental contaminants are widespread due to long-distance transport by atmospheric and ocean currents. The chemical compounds accumulate and biomagnifies in the marine food web and humans. The Arctic populations have the highest levels of some persistent organic pollutants (POPs) globally [1]. High serum POP levels in humans in the Arctic area have been related to age, marine food intake and smoking rate [2]. The toxic and immunomodulatory effects of POPs have previously been investigated, especially in relation to polychlorinated biphenyls (PCBs), organochlorine pesticides (OCPs) and perfluoroalkylated substances (PFASs) [3–5]. In addition, previous studies have reported the effects of POP exposure on the immune system in laboratory animals and humans, especially in connection with depressed immunity and reduced vaccination response [6–12]. Several biomarkers have been investigated in relation to POP exposure, in e.g. blood, urine, hair, cord blood, breast milk and breath [13]. However, exposure to POPs during pregnancy can have an impact on the foetal development, since POPs are present in the maternal circulation and can cross the placenta [14]. Furthermore, the newborn child is exposed to POPs via breastfeeding. The immunotoxic POP exposure in prenatal and early postnatal life is important to investigate, since the immune system develops extensively in this period of life [15].

Additionally, the status of the mother’s immune system during pregnancy in relation to POP levels is important to investigate.

In the last 5 years (2011–2016), the total number of registered births per year in Greenland has been approximately 800 [16]. Maternal health in Greenland is a complex issue, challenged by long distances, sparse infrastructure, fluctuating health care staff and centralised hospitals in the larger towns. Greenlandic pregnant women are offered prenatal visits and at least 90% receive complete prenatal care [17]. In the case of high-risk pregnancies or complicated childbirths, women from remote areas are transferred to Queen Ingrid Hospital (QIH) in Nuuk.

To evaluate the long-term health effects of environmental contaminant exposure, it is essential to examine the current health aspect in pregnant women in the Arctic area. The aim of the present study was to investigate possible associations between exposure to POPs and status of haematological markers in Greenlandic pregnant women.

## Materials and methods

### Study population

**Figure 1.**
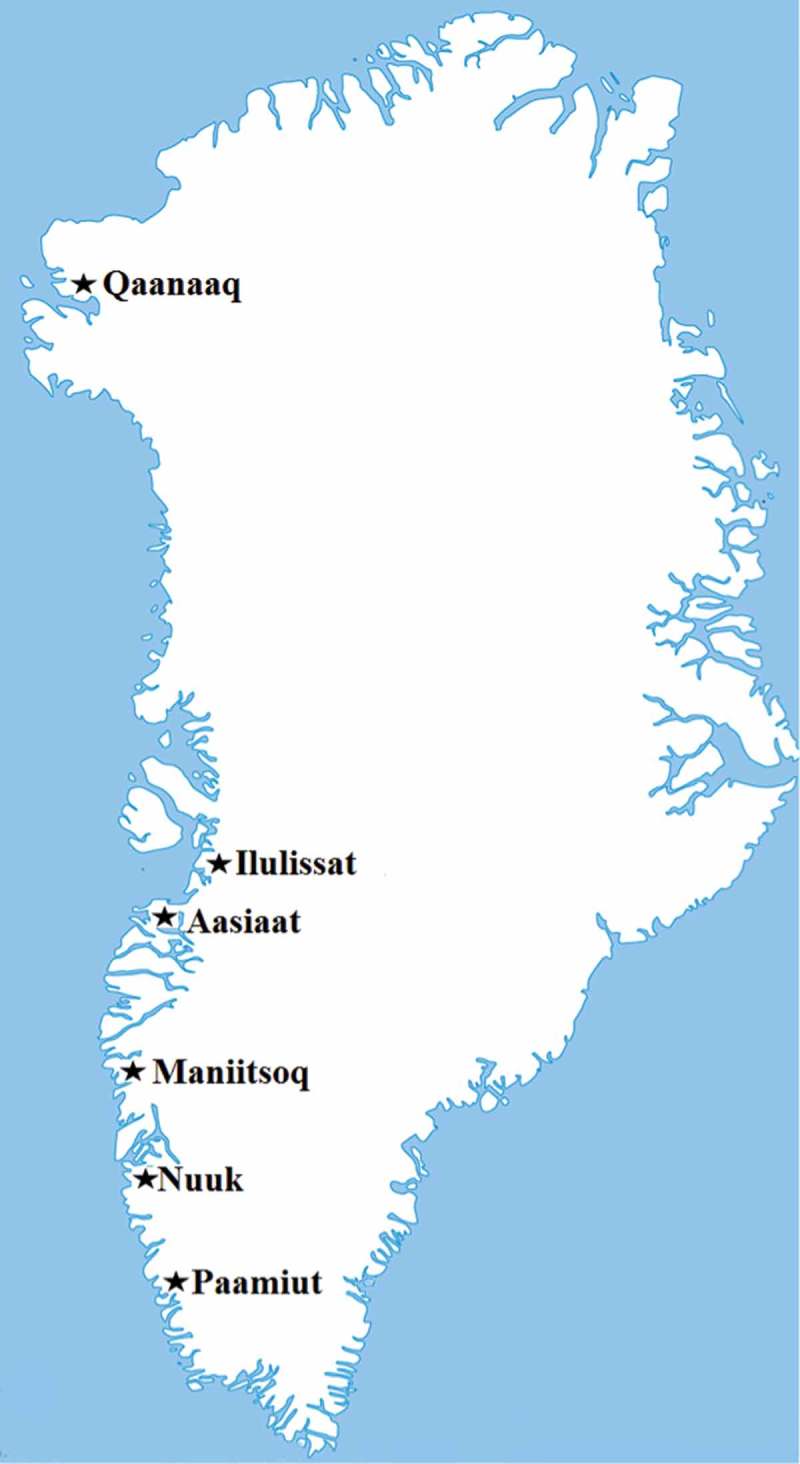
Map of Greenland showing the six selected town regions for the ACCEPT sub-study in 2010-11. Participants were enrolled in Qaanaaq, Ilulissat, Aasiaat, Maniitsoq, Nuuk, and Paamiut [18, 19].

**Figure 2.**
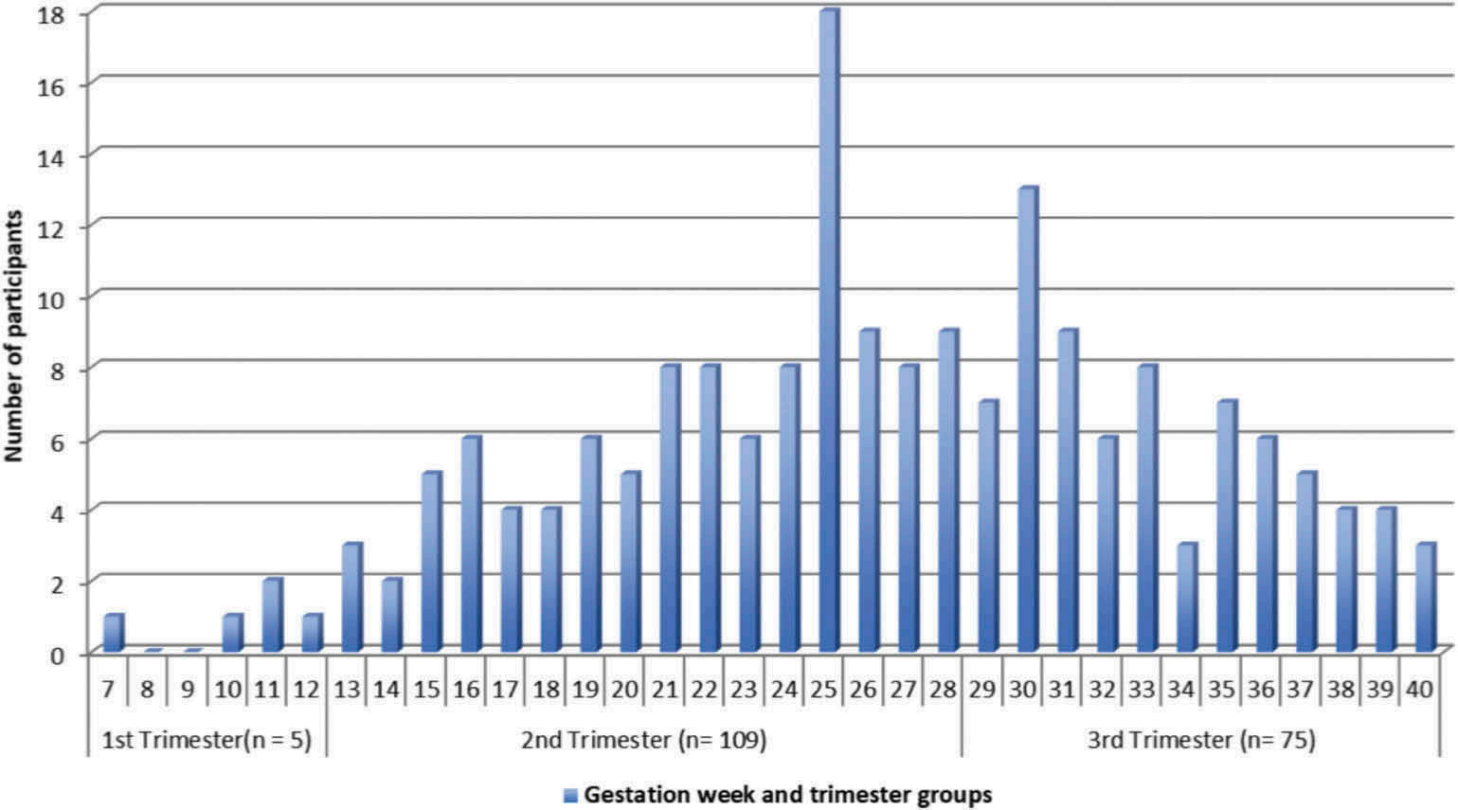
The total number (*N* = 189) of participants’ data stratified by gestation week and trimester groups at inclusion time.

The present study is a cross-sectional sub-study of the geographical and prospective Greenlandic Birth cohort Adaption to Climate Change, Environmental Pollution and Dietary Transition (ACCEPT) established during 2010–2015. The ACCEPT project focuses on the lifestyle, diet, environmental contaminant exposure and maternal health during pregnancy and health and development of the children. The detailed methods for collection data regarding the lifestyle, reproductive factors, food intake and measuring levels of POPs during 2010–2011 have been described elsewhere [18,19]. Enrolment was conducted during 2010–2011 at six selected region towns at the Greenlandic West coast (Figure 1); 192 pregnant women were enrolled. The participants were included during gestation week (GW) 7–40 (Figure 2), by a doctor employed at QIH. With the exception from Nuuk, enrolment depended on coast visit carried out by the doctor.

Thus, a convenient sampling method was used to cover different geographical parts of Greenland. The inclusion criteria were: ≥18 years of age, had lived for 50% or more of their life in Greenland, minimum one parent from Greenland, and “expected normal pregnancies”. Three participants enrolled in Nuuk were excluded, due to non-Inuit descent (no parents from Greenland) and living >50% of their life in Denmark.

Finally, the total study population consisted of 189 Greenlandic pregnant women expected to be a representative group for pregnant women in the included towns. At inclusion, the participants completed a food frequency questionnaire regarding intake of traditional and imported food, and a lifestyle questionnaire with baseline information (age, pre-pregnancy BMI, self-reported smoking status, alcohol intake, etc.) [18].

### Haematological markers

At inclusion, venous blood was drawn into dry tubes and Vacutainer tubes containing trisodium ethylenediaminetetraacetic acid (EDTA) (BD Vacutainer®, UK). Routine laboratory analysis methods were performed and 24 haematological markers (for abbreviations please see Table 2) were analysed at The Central Laboratory at QIH. The laboratory tests were analysed at Sysmex XT-1800i (Sysmex Canada, Inc., Mississauga, Ontario, Canada), Architect 2000 (Abbott Diagnostics, Abbott Park, IL, USA) and Architect c8000 (Abbott Diagnostics, Abbott Park, IL, USA). All tests were performed according to the manufacturers’ specifications and laboratory standards. For participants included in Nuuk, the blood samples were analysed immediately after enrolment. For inclusion of participants from remote districts, and hence all towns except from Nuuk, the blood samples were stored at +4°C and subsequently (between 1 and 8 days) transported to The Central Laboratory at QIH for analysis.

**Table 1.** Baseline characteristics for study population (*N* = 189).

Parameters	
Age at inclusion (years)	27.00 (18–42)
Pre-pregnancy BMI^a^ (kg/m^2^)	24.24 (16.38–46.57)
Parity^b^, *n* (%)	189 (100)
0	69 (36.5)
1–2	94 (49.7)
≥3	26 (13.8)
Smoking status, *n* (%)^c^	188 (100)
Current	87 (46.3)
Non-current	101 (53.7)
P-cotinine^d^ (ng/ml)	0.500 (0.500–190.5)
Current (median)	75.7
Non-current (median)	0.5
Alcohol during pregnancy, *n* (%)^c^	185 (100)
No (<1 a month)	179 (97)
Yes (>1 a month)	6 (3)
Iron substitution, *n* (%)	189 (100)
No	32 (16.9)
Yes	157 (83.1)
n-3/n-6 fatty acid ratio	0.22 (0.08–0.87)
Collection site, *n* (%)	189 (100)
Nuuk	92 (48.7)
Maniitsoq	39 (20.6)
Ilulissat	33 (17.5)
Aasiaat	18 (9.5)
Paamiut	4 (2.1)
Qaanaaq	3 (1.6)
Level of POPs^e^	
sumPCBs^f^ (μg/kg lipid)	189.2 (55.70–2714)
sumOCPs^g^ (μg/kg lipid)	240.5 (41.10–2198)
sumPFASs^h^ (ng/mL)	18.5 (6.14–96.43)

*N*: total number of participants in the study population.

*n* (%): number and per cent of participants in the groups and with the corresponding answer.

^a^BMI: Body mass index in kg/m^2^.

^b^Parity: number of full term pregnancies.

^c^Missing answer(s), not included in the per cent calculation.

^d^P-cotinine: plasma-cotinine in ng/ml.

^e^POPs: persistent organic pollutants.

^f^sumPCBs: sum of 14 polychlorinated biphenyls.

^g^sumOCPs: sum of 11 organochlorine pesticides.

^h^sumPFASs: sum of 15 perfluoroalkylated substances.

For single POP congeners and the sum of n-3 and n-6 fatty acids, see reference 19.

Data are presented as median (min – max), unless otherwise indicated.

**Table 2.** Baseline information of the haematological markers for the study population (*N* = 189).

Parameter	Abbreviation	SI unit	*n*	Median	Min – Max	General reference range
Haemoglobin	Hgb	mmol/L	168	7.2	5.6–8.8	7.0–9.4^a^
Haematocrit	Hct	ratio (%)^f^	168	0.39	0.27–0.49	0.35–0.47^a^
Glycated haemoglobin A1c	HbA1c	mmol/mol	148	25.5	13.8–44.6	31–44^a^
Basophil count	BASO	10^9^/L	168	0.02	0.01–0.16	0.0–0.20 ^b^
Eosinophil count	EO	10^9^/L	162	0.10	0.00–0.81	0.0–0.50 ^b^
Lymphocyte count	LYMPH	10^9^/L	160	1.57	0.26–3.14	0.70–4.80 ^b^
Monocyte count	MONO	10^9^/L	162	0.39	0.02–1.22	0.0–1.10 ^b^
Neutrophil count	NEU	10^9^/L	160	5.43	0.71–11.70	1.80–7.40 ^b^
Mean corpuscular haemoglobin	MCH	fmol/L	168	1.9	1.4–2.1	1.7–2.1^a^
Mean corpuscular haemoglobin concentration	MCHC	mmol/L	168	18.4	15.8–22.3	18.6–22.3^a^
Mean erythrocyte corpuscular volume	MCV	fL^g^	168	100	78–119	81–109^a^
Mean platelet volume	MPV	fL^g^	168	10.3	7.4–13.3	6.4–11.00^c^
Platelet distribution width	PDW	fL^g^	168	11.8	7.4–18.5	8.9–15.5^d^
Plateletcrit	PCT	ratio (%)^h^	168	0.27	0.09–0.66	0.13–0.34^d^
Platelet count (thrombocyte count)	PLT	10^9^/L	168	258	85–653	135–400^a^
Platelet – larger cell ratio	P-LCR	ratio (%)^i^	168	0.272	0.07–0.497	–
Red blood cell count	RBC	10^12^/L	168	3.89	2.89–5.20	3.66–5.10^a^
Red cell distribution width (standard deviation)	RDW-SD	fL^g^	168	49.9	38.7–98.3	39.0–46.0 ^5^
Red cell distribution width (cell volume)	RDW-CV	ratio(%)^j^	168	0.142	0.118–0.262	0.116–0.146^e^
White blood cell count (leukocyte count)	WBC	10^9^/L	168	7.5	1.6–15.1	3.0–8.5^a^
Thyroid stimulating hormone	TSH	mIU/L	132	0.84	0.01–4.41	0.4–3.8^a^
Thyroxine (total)	TT_4_	nmol/L	131	120	81–240	60–140^a^
Triiodothyronine (total)	TT_3_	nmol/L	130	2.40	1.54–4.11	1.10–2.38^a^
Uric acid	URIC	mmol/L	169	0.23	0.11–0.40	0.15–0.35^a^

*N*: total number of participants in the study population. *n*: number of participants with data to the corresponding parameter.

Missing data: *N* – *n*.

General reference range listed with reference to: ^a^local recommendation for the Central Laboratory at Queen Ingrid Hospital, Greenland,

^b^[22], ^c^[23], ^d^[24], ^e^[25].

^f^Hct ratio: volume fraction of MCV and RBC in %.

^g^fL: 10–15 L.

^h^PCT ratio: platelet count in the blood as a percentage to the whole blood volume.

^i^P-LCR ratio: the percentage of the platelets with a size of more than 12 fL.

^j^RDW-CV ratio: RDW-SD/MCV.

“–“: no given reference range.

The data are presented in The International System (SI) unit with a median value and minimum – maximum. A few haematological markers were not available for some participants, due to missing data or laboratory measurement errors. The Central Laboratory at QIH provided the general reference ranges for the given parameters for non-pregnant, non-Inuit adults, which were in accordance with the current literature laboratory ranges [20,21].

When the reference range was not given from The Central Laboratory at QIH, the parameter was listed with the reference range to several relevant laboratory studies; 1) a study including Danish pregnant women [22]; 2) a database review for maternal laboratory studies [23]; 3) a study regarding platelet markers in pregnant women [24]; and 4) a reference range for RDW for healthy adults [25]. For BASO, EO, LYMPH, MONO, and NEU count, the data were presented in 109/L. The SI-unit for HbA1c was analysed at the Central Laboratory at QIH in the unit % and was recalculated to mmol/mol using the formula: ((HbA1c (%) – 2.15)/0.0915), due to new recommendations for HbA1c [26].

### POP variables

Serum blood samples were analysed for 14 polychlorinated biphenyls (sumPCBs, µg/kg lipid), 11 organochlorine pesticides (sumOCPs, µg/kg lipid) at Centre de Toxicologie, Institute National de Santé Publique du Québec, and 15 perfluoroalkylated substances (sumPFASs, ng/mL) at the Department of Environmental Science, Aarhus University. For the details of measurement and for the specific compounds see Ref. 19.

#### Plasma-cotinine

Plasma-cotinine (P-cotinine), an active metabolite of nicotine, determined as a predictor of recent tobacco smoking, was analysed at the Centre of Arctic Health & Molecular Epidemiology, Aarhus University, in ng/ml. The detection limit was 1 ng/ml. Cotinine values less than 1 ng/ml were given as 0.500 ng/ml in the statistical analysis.

### Plasma fatty acids

Plasma fatty acids including n-3 polyunsaturated fatty acids and n-6 fatty acids were determined by capillary gas-liquid chromatography at the Biology Department, University of Guelph, Canada. The ratio between n-3 and n-6 is known to be a strong indicator of marine food intake. For the detailed description of fatty acids, see Ref. 19.

### Statistical analysis

All statistical analyses were performed in SPSS Statistics version 22.0 (SPSS Inc. Chicago, IL, USA). The data distribution was checked by Q-Q-plots. When data on the haematological markers were non-normally distributed, natural logarithmic (ln) transformation improved the normality, and the analyses were performed on the ln-transformed data. Regarding the POP variables (sumPCBs, sumOCPs and sumPFASs), all the statistical analyses were performed on ln-transformed data. The associations between haematological markers and the POP variables (sumPCBs, sumOCPs and sumPFASs) were assessed using a linear regression model under adjustment for age, pre-pregnancy BMI, parity, gestation week (GW), P-cotinine level and alcohol intake. The change in the estimate principle was used to identify the potential confounders, given that the change in estimates was more than 5% (Greenland 1989). Data for the linear regression model are presented by crude and adjusted statistics with a β-coefficient, 95% confidence interval (95% CI) and p-value. Considering the influence of polyunsaturated fatty acids on the immune system, the linear regression analysis was further adjusted for the n-3/n-6 ratio together with the other mentioned confounders. The statistical significant level was set to *p* ≤ 0.05.

Supplementary Table 1 presents the haematological markers in relation to the general reference range and trimester groups. Pregnant women in 1st trimester (GW ≤ 12) counted only five participants, thus 1st and 2nd trimesters were merged. Supplementary Table 2 shows the n-3/n-6 ratio, total lipid and levels of POPs in relation to the trimester groups. To check the difference between the continuous variables in the trimester groups, an independent t-test was used. For further explanation, please see the Supplementary material.

### Ethics

Each participating woman gave informed consent prior to inclusion. ACCEPT was approved by the Ethical Committee in Greenland in accordance with the Helsinki declaration II.

## Results

### Study population

Table 1 shows the baseline characteristics for the study population. The median age was 27 years and the median pre-pregnancy body mass index (BMI) was 24.24 kg/m2. For parity, defined as the number of full term pregnancies, approximately 50% of the participants had 1–2 children. Forty-six per cent were current smokers. The current smokers had a higher plasma cotinine level than non-current smokers (median: 75.7 ng/ml vs. 0.5 ng/ml, *p* < 0.0001). Only a few participants (3%) stated to drink alcohol during their pregnancy; 83% substituted with daily oral iron supplement, which is an important recommendation to prevent maternal anaemia. The median n-3/n-6 ratio was 0.22.

Approximately 50% of the participants were enrolled in the capital Nuuk. The median POP levels for sumPCBs and sumOCPs were 189.2 and 240.5 µg/kg lipid, respectively, while for sumPFASs it was 18.5 ng/ml (Table 1).

### Haematological markers

Table 2 presents the baseline information for the 24 analysed haematological markers and their abbreviation. The haematological marker’s median values were within the general reference range for a non-pregnant, non-Inuit adult, although some of the participants had median values of the given haematological markers either below or above the general reference range: HbA1c (lower), MCHC (lower), RDW-SD (higher) and TT3 (higher) (Table 2).

### Association between haematological markers and POP variables

In Tables 3, 4 and 5, the data for the linear regression analyses are shown for association between the haematological markers and the sum of the three POP variables (sumPCBs, sumOCPs and sumPFASs), respectively. The haematological markers EO, LYMPH, NEU and WBC were significant and inversely associated with sumPCBs (Table 3), sumOCPs (Table 4) and sumPFASs (Table 5) both before and after adjustment for confounders (age, pre-pregnancy BMI, parity, gestation week (GW), P-cotinine level and alcohol intake). In addition, for sumPFASs (Table 5), a significant inverse association was found for the markers MONO, MCHC, PCT and PLT. Moreover, a significant positive association was observed for sumPFASs and the markers Hct and MCV (Table 5). For MPV, PDW, P-LCR, RDW-SD and URIC (borderline), the associations were significant for the crude data for sumPFASs, but the significant association disappeared after adjustment for the confounders (Table 5).

**Table 3.** The association between haematological markers and serum level of sumPCBs (*N* = 189).

	Crude			Adjusted^a^			Adjusted^b^		
Parameter	*β*	(95%CI)	*p*	*β*	(95%CI)	*p*	*β*	(95%CI)	*p*
Hgb (mmol/L)	0.02	(−0.11;0.16)	*0.73*	0.05	(−0.09;0.18)	*0.51*	0.03	(−0.12;0.18)	*0.69*
Hct (ratio)	0.00	(−0.01;0.01)	*0.81*	0.00	(−0.01;0.01)	*0.94*	0.00	(−0.01;0.01)	*0.88*
HbA1c(mmol/mol)^c^	−0.03	(−0.07;0.02)	*0.21*	−0.03	(−0.08;0.01)	*0.12*	−0.04	(−0.08;0.01)	*0.13*
BASO (10^9^/L)^c^	0.09	(−0.04;0.23)	*0.18*	0.07	(−0.07;0.22)	*0.32*	0.09	(−0.08;0.25)	*0.30*
EO (10^9^/L)^c^	−0.24	(−0.39;-0.08)	***<0.01***	−0.20	(−0.36;-0.04)	***0.01***	−0.17	(−0.34;0.01)	0.06*
LYMPH (10^9^/L)	−0.12	(−0.23;-0.02)	***0.02***	−0.13	(−0.24;-0.01)	***0.03***	−0.11	(−0.24;0.01)	0.08*
MONO (10^9^/L)^c^	−0.09	(−0.21;0.03)	*0.14*	−0.09	(−0.20;0.03)	*0.13*	−0.11	(−0.24;0.01)	0.07*
NEU (10^9^/L)^c^	−0.19	(−0.30;-0.07)	***<0.01***	−0.18	(−0.29;-0.07)	***<0.01***	−0.17	(−0.29;-0.04)	***0.01***
MCH (fmol/L)	0.02	(−0.01;0.04)	*0.16*	0.01	(−0.01;0.04)	*0.30*	0.01	(−0.02;0.04)	*0.59*
MCHC (mmol/L)	0.00	(−0.35;0.35)	*0.99*	0.09	(−0.28;0.45)	*0.64*	0.03	(−0.37;0.43)	*0.88*
MCV (fL)	0.87	(−1.24;2.99)	*0.41*	0.16	(−2.01;2.34)	*0.88*	0.09	(−2.29;2.47)	*0.94*
MPV (fL)	−0.04	(−0.28;0.20)	*0.72*	−0.17	(−0.41;0.08)	*0.17*	−0.23	(−0.50;0.04)	0.09*
PDW (fL)	−0.18	(−0.68;0.32)	*0.47*	−0.41	(−0.93;0.11)	*0.12*	−0.54	(−1.11;0.02)	0.06*
PCT (ratio)^c^	−0.02	(−0.07;0.04)	*0.54*	0.00	(−0.07;0.06)	*0.89*	0.02	(−0.04;0.09)	*0.49*
PLT (10^9^/L)^c^	−0.02	(−0.09;0.05)	*0.53*	0.00	(−0.07;0.08)	*0.94*	0.04	(−0.04;0.11)	*0.37*
P-LCR (ratio)	0.00	(−0.02;0.02)	*0.78*	−0.01	(−0.03;0.01)	*0.23*	−0.02	(−0.04;0.00)	*0.11*
RBC (10^12^/L)	−0.02	(−0.10;0.06)	*0.58*	0.00	(−0.08;0.08)	*0.96*	0.00	(−0.08;0.09)	*0.93*
RDW-SD (fL)^c^	0.00	(−0.04;0.04)	*0.93*	−0.01	(−0.05;0.03)	*0.62*	0.00	(−0.04;0.04)	*0.99*
RDW-CV (ratio)^c^	−0.01	(−0.04;0.01)	*0.25*	−0.01	(−0.04;0.01)	*0.29*	0.00	(−0.03;0.03)	*0.84*
WBC (10^9^/L)	−0.76	(−1.33;-0.18)	***0.01***	−0.79	(−1.38;-0.20)	***0.01***	−0.72	(−1.37;-0.08)	***0.03***
TSH (mIU/L)^c^	−0.06	(−0.22;0.11)	*0.50*	−0.05	(−0.22;0.13)	*0.59*	−0.09	(−0.28;0.10)	*0.36*
TT_4_ (nmol/L)^c^	−0.01	(−0.06;0.04)	*0.65*	−0.02	(−0.08;0.03)	*0.36*	−0.03	(−0.08;0.03)	*0.36*
TT_3_ (nmol/L)^c^	−0.01	(−0.06;0.04)	*0.64*	−0.03	(−0.08;0.02)	*0.25*	−0.03	(−0.09;0.02)	*0.21*
URIC(mmol/L)	0.01	(−0.01;0.02)	*0.27*	0.00	(−0.01;0.02)	*0.47*	0.01	(−0.01;0.02)	*0.50*

*N*: total number of participants in the study population. sumPCBs: ln-transformed data with the sum of 14 polychlorinated biphenyls.

For single POP congeners, see Ref. 19.

^a^Linear regression model adjusted for: age, pre-pregnancy BMI, parity, gestation week (GW), P-cotinine and alcohol intake during pregnancy.

^b^Linear regression model adjusted for: age, pre-pregnancy BMI, parity, gestation week (GW), P-cotinine, alcohol intake during pregnancy and n-3/n-6 ratio.

^c^ln-transformed haematological marker in the statistical analysis.

*borderline significant.

*β*: linear regression coefficient.

CI: confidence interval.

**Table 4.** The association between haematological markers and serum level of sumOCPs (*N* = 189).

	Crude			Adjusted^a^			Adjusted^b^		
Parameter	*β*	(95%CI)	*p*	*β*	(95%CI)	*p*	*β*	(95%CI)	*p*
Hgb (mmol/L)	0.06	(−0.06;0.18)	*0.33*	0.06	(−0.05;0.18)	*0.28*	0.06	(−0.07;0.19)	*0.35*
Hct (ratio)	0.00	(0.00;0.01)	*0.30*	0.00	(−0.01;0.01)	*0.53*	0.00	(−0.01;0.01)	*0.43*
HbA1c(mmol/mol)^c^	−0.01	(−0.05;0.03)	*0.58*	−0.02	(−0.06;0.01)	*0.21*	−0.02	(−0.06;0.02)	*0.24*
BASO (10^9^/L)^c^	0.05	(−0.07;0.17)	*0.41*	0.05	(−0.07;0.18)	*0.42*	0.06	(−0.08;0.20)	*0.41*
EO (10^9^/L)^c^	−0.19	(−0.32;-0.05)	***0.01***	−0.14	(−0.28;-0.01)	***0.04***	−0.11	(−0.26;0.04)	*0.17*
LYMPH (10^9^/L)	−0.11	(−0.20;-0.01)	***0.02***	−0.11	(−0.21;-0.01)	***0.03***	−0.10	(−0.21;0.01)	0.08*
MONO (10^9^/L)^c^	−0.08	(−0.18;0.02)	*0.13*	−0.06	(−0.16;0.04)	*0.25*	−0.08	(−0.18;0.03)	*0.17*
NEU (10^9^/L)^c^	−0.14	(−0.24;-0.04)	***0.01***	−0.13	(−0.23;-0.03)	***0.01***	−0.11	(−0.22;0.00)	***0.05***
MCH (fmol/L)	0.01	(−0.02;0.03)	*0.59*	0.00	(−0.02;0.03)	*0.71*	0.00	(−0.03;0.02)	*0.86*
MCHC (mmol/L)	−0.06	(−0.36;0.24)	*0.71*	0.03	(−0.28;0.34)	*0.86*	−0.03	(−0.37;0.31)	*0.93*
MCV (fL)	0.52	(−1.32;2.35)	*0.58*	−0.03	(−1.89;1.83)	*0.98*	−0.14	(−2.18;1.90)	*0.90*
MPV (fL)	0.01	(−0.19;0.22)	*0.90*	−0.10	(−0.31;0.11)	*0.35*	−0.14	(−0.37;0.09)	*0.23*
PDW (fL)	−0.06	(−0.50;0.37)	*0.77*	−0.27	(−0.71;0.18)	*0.24*	−0.36	(−0.85;0.12)	*0.14*
PCT (ratio)^c^	−0.02	(−0.07;0.03)	*0.45*	−0.01	(−0.07;0.04)	*0.58*	0.01	(−0.05;0.06)	*0.68*
PLT (10^9^/L)^c^	−0.03	(−0.08;0.03)	*0.39*	−0.01	(−0.07;0.05)	*0.73*	0.01	(−0.05;0.08)	*0.43*
P-LCR (ratio)	0.00	(−0.01;0.02)	*0.83*	−0.01	(−0.02;0.01)	*0.44*	−0.01	(−0.03;0.01)	*0.27*
RBC (10^12^/L)	0.02	(−0.05;0.09)	*0.52*	0.03	(−0.04;0.09)	*0.42*	0.04	(−0.03;0.12)	*0.28*
RDW-SD (fL)^c^	0.00	(−0.04;0.03)	*0.82*	−0.01	(−0.04;0.02)	*0.54*	0.00	(−0.04;0.03)	*0.90*
RDW-CV (ratio)^c^	−0.01	(−0.03;0.01)	*0.24*	−0.01	(−0.04;0.01)	*0.25*	0.00	(−0.03;0.02)	*0.75*
WBC (10^9^/L)	−0.61	(−1.11;-0.11)	***0.02***	−0.61	(−1.12;-0.11)	***0.02***	−0.54	(−1.10;-0.01)	0.06*
TSH (mIU/L)^c^	−0.07	(−0.21;0.08)	*0.36*	−0.05	(−0.20;0.10)	*0.51*	−0.09	(−0.25;0.07)	*0.27*
TT_4_ (nmol/L)^c^	0.00	(−0.05;0.04)	*0.82*	−0.01	(−0.06;0.03)	*0.56*	−0.02	(−0.07;0.03)	*0.51*
TT_3_ (nmol/L)^c^	0.00	(−0.04;0.04)	*0.94*	−0.02	(−0.06;0.03)	*0.45*	−0.02	(−0.07;0.03)	*0.38*
URIC(mmol/L)	0.00	(−0.01;0.01)	*0.36*	0.00	(−0.01;0.01)	*0.44*	0.00	(−0.01;0.02)	*0.45*

*N*: total number of participants in the study population. sumOCPs: ln-transformed data with the sum of 11 organochlorine pesticides.

For single POP congeners, see [19].

^a^Linear regression model adjusted for: age, pre-pregnancy BMI, parity, gestation week (GW), P-cotinine and alcohol intake during pregnancy.

^b^Linear regression model adjusted for: age, pre-pregnancy BMI, parity, gestation week (GW), P-cotinine, alcohol intake during pregnancy and n-3/n-6 ratio.

^c^ln-transformed haematological marker in the statistical analysis.

*borderline significant.

*β*: linear regression coefficient.

CI: confidence interval.

**Table 5.** The association between haematological markers and serum level of sumPFASs (*N* = 189).

	Crude			Adjusted^a^			Adjusted^b^		
Parameter	*β*	(95%CI)	*p*	*β*	(95%CI)	*p*	*β*	(95%CI)	*p*
Hgb(mmol/L)	−0.02	(−0.20;0.17)	*0.87*	0.01	(−0.18;0.21)	*0.88*	−0.02	(−0.24;0.18)	*0.80*
Hct(ratio)	0.01	(0.00;0.03)	***0.02***	0.01	(0.00;0.03)	***0.04***	0.02	(0.00;0.03)	***0.04***
HbA1c(mmol/mol)^c^	−0.02	(−0.07;0.04)	*0.62*	−0.01	(−0.06;0.05)	*0.86*	0.00	(−0.07;0.06)	*0.97*
BASO(10^9^/L)^c^	0.07	(−0.10;0.25)	*0.41*	0.05	(−0.15;0.25)	*0.60*	0.07	(−0.15;0.29)	*0.53*
EO(10^9^/L)^c^	−0.32	(−0.53;-0.12)	***<0.01***	−0.22	(−0.44; 0.01)	0.06*	−0.16	(−0.40;0.09)	0.21**
LYMPH(10^9^/L)^c^	−0.26	(−0.40;-0.12)	***<0.01***	−0.28	(−0.44;-0.13)	***<0.01***	−0.30	(−0.47;-0.13)	***<0.01***
MONO(10^9^/L)^c^	−0.24	(−0.40;-0.09)	***<0.01***	−0.27	(−0.42;-0.11)	***<0.01***	−0.29	(−0.46;-0.13)	***<0.01***
NEU(10^9^/L)^c^	−0.27	(−0.42;-0.12)	***<0.01***	−0.25	(−0.41;-0.09)	***<0.01***	−0.21	(−0.39;-0.04)	***0.02***
MCH(fmol/L)	0.01	(−0.02;0.05)	*0.44*	0.00	(−0.03;0.04)	*0.85*	−0.01	(−0.05;0.03)	*0.54*
MCHC(mmol/L)	−0.78	(−1.22;-0.34)	***<0.01***	−0.70	(−1.18;-0.23)	***<0.01***	−0.87	(−1.39;-0.35)	***<0.01***
MCV(fL)	4.74	(2.04;7.44)	***<0.01***	3.79	(0.89;6.68)	***0.01***	3.73	(0.56;6.90)	***0.02***
MPV(fL)	0.41	(0.10;0.72)	***<0.01***	0.22	(−0.11;0.56)	0.19**	0.16	(−0.21;0.52)	0.40**
PDW(fL)	0.71	(0.07;1.36)	***0.03***	0.36	(−0.35;1.07)	0.32**	0.25	(−0.53;1.03)	0.52**
PCT(ratio)^c^	−0.14	(−0.21;-0.07)	***<0.01***	−0.13	(−0.21;-0.05)	***<0.01***	−0.10	(−0.19;-0.01)	***0.03***
PLT(10^9^/L)^c^	−0.18	(−0.26;-0.09)	***<0.01***	−0.15	(−0.25;-0.05)	***<0.01***	−0.11	(−0.21;-0.004)	***0.04***
P-LCR(ratio)	0.03	(0.01;0.05)	***0.01***	0.02	(−0.01;0.04)	0.19**	0.01	(−0.02;0.04)	*0.42*
RBC(10^9^/L)^c^	−0.04	(−0.14;0.07)	*0.50*	0.00	(−0.11;0.11)	*0.99*	0.01	(−0.11;0.13)	*0.83*
RDW-SD(fL)^c^	0.05	(0.01;0.10)	***0.03***	0.04	(−0.01;0.10)	0.10**	0.06	(0.00;0.12)	***0.04***
RDW-CV(ratio)^c^	0.00	(−0.03;0.03)	*0.88*	0.00	(−0.03;0.04)	*0.88*	0.02	(−0.02;0.06)	*0.31*
WBC(10^9^/L)^c^	−1.56	(−2.30;-0.83)	***<0.01***	−1.63	(−2.40;-0.86)	***<0.01***	−1.56	(−2.41;-0.70)	***<0.01***
TSH(mIU/L)^c^	−0.12	(−0.39;0.16)	*0.41*	−0.26	(−0.56;0.04)	*0.08*	−0.35	(−0.67;-0.03)	***0.03***
TT_4_(nmol/L)	−0.01	(−0.08;0.06)	*0.72*	−0.03	(−0.11;0.05)	*0.43*	−0.04	(−0.12;0.05)	*0.39*
TT_3_(nmol/L)	−0.02	(−0.08;0.05)	*0.65*	−0.03	(−0.11;0.04)	*0.41*	−0.04	(−0.12;0.05)	*0.39*
URIC(mmol/L)	0.01	(−0.002;0.03)	0.09*	0.01	(−0.01;0.03)	0.30**	0.01	(−0.01;0.03)	0.39**

*N*: total number of participants in the study population. sumPFASs: ln-transformed data with the sum of 15 perfluoroalkylated substances. For single POP congeners, see Ref. 19.

^a^Linear regression model adjusted for: age, pre-pregnancy BMI, parity, gestation week (GW), P-cotinine and alcohol intake during pregnancy.

^b^Linear regression model adjusted for: age, pre-pregnancy BMI, parity, gestation week (GW), P-cotinine, alcohol intake during pregnancy and n-3/n-6 ratio.

^c^ln-transformed haematological marker in the statistical analysis.

*borderline significant.

**p-value significant in crude data material, but non-significant after adjustment.

*β*: linear regression coefficient.

CI: confidence interval.

Further adjustment for the n-3/ n-6 fatty acid ratio resulted in slight attenuation of the significant associations between haematological markers and PCBs and OCPs (Tables 3 and 4), while the significant associations between PFASs and haematological markers still exist (Table 5).

### Supplementary materials

Few haematological markers were significantly different between the two trimester groups (1st and 2nd trimester vs. 3rd trimester) (supplementary Table 1), and pregnant women in 3rd trimester had a significantly higher level of the total lipid, compared to women in the 1st and 2nd trimester groups (supplementary Table 2). For further explanation, see “Supplementary material”.

Supplementary_material.docx

## Discussion

The present ACCEPT sub-study (2010–2011) found several significant associations between the sum of POPs and haematological markers in Greenlandic pregnant women. Some of the median values for the 24 analysed haematological markers were not within the given general reference ranges for non-pregnant, non-Inuit adults. This suggests a comprehensive variation within the general reference ranges of different haematological markers, and that pregnancy might affect the markers. As presented, we found that sumPCBs, sumOCPs and sumPFASs inversely affected EO, LYMPH, NEU and WBC. These specific haematological biomarkers are all parts of the immunological system and included in the blood cell counts. In addition, we found a significant inverse association between sumPFASs and the MONO, MCHC, PCT and PLT markers. Furthermore, we found a significant positive association between sumPFASs and the markers Hct and MCV.

The overall function of the immune system during pregnancy is to protect the organism against infections. If the immunological response is affected, e.g. by POPs, the clinical influence might be an impaired maternal health. This may cause susceptibility for infections, preeclampsia or thrombocytopenia in the mother, and ultimately can have foetal consequences in relation to abortus habitualis, intrauterine growth retardation and preterm delivery [27,28]. Furthermore, the prenatal exposure to POPs, especially PFASs, can lead to suppressed immune responses in early childhood [7–11,29,30].

To our knowledge, this study is the very first to report on associations between POP exposure and haematological markers in Greenlandic pregnant women. There are few reports on the association between environmental contaminant exposure and haematological markers [31–33]. The literature on this specific topic for pregnant women is sparse, however, the limited literature suggests an immunosuppression measured through specific biomarkers in humans exposed to environmental contaminants. The levels of POPs have previously been found to be high in the given study population in Greenland [19], which indicates the importance to investigate the POP effect on human health, e.g. by examining new biomarkers. Hansen et al. [34] investigated exposure to organochlorines (OC) including PCBs and OCPs in relation to pregnant women in 2nd trimester and postpartum (day 3 and 6 weeks). The Hansen et al. study showed that wet-weight OC levels peaked at birth, but not upon lipid adjustment, suggesting a physiological lipid change in pregnancy and postpartum. Similarly, in our present study population, we observed a higher plasma total lipid in the samples collected in the 3rd trimester compared to those collected in 1st and 2nd trimesters, and neither the lipid-adjusted PCB and OCP levels nor PFAS levels differ between trimester groups (supplementary Table 2). Furthermore, a previous study has explored the connection between POP levels and several blood cell counts in children (age 18 month and follow-up at age 5 years), and the results showed that prenatal exposure to OCPs was marginally associated with decreases in neutrophil counts [29]. In addition, a Norwegian study investigated several immunomodulatory genes (e.g. CYTL1, IL27) associated with PFAS exposure, and it showed toxic effects for the immunological and developmental functions [30].

Due to physiological changes and general maternal health, the haematological markers change during pregnancy [22,35]. Moreover, the haematological markers are an important diagnostic tool, e.g. to monitor the mother’s wellbeing and to secure the development of the foetus. Therefore, the physiological changes are an important perquisite for an uncomplicated pregnancy. To cope with the large blood flow to organs and placenta, the blood volume is expanded from approximately 4 to 6 L, though with individual differences [21] and this affects the erythrocyte volume and haemoglobin concentration. Besides the hemodilution, the glomerular filtration rate increases, the regulation of glucose is also affected, and the thyroid hormone levels alter during pregnancy with an increase in the beginning of the pregnancy and a decrease at the 3rd trimester [20,21,36]. In addition, the mother’s immune system is changed during pregnancy, to protect the foetus from improper immune reactions, e.g. by increasing the number of T-cells and leukocytes in the peripheral blood flow [21]. All these physiological changes are, as mentioned, very individual. In perspective, the physiological aspects are yet to be investigated in relation to POP exposure, e.g. how the contaminants might affect the placentation or changes in the placenta barrier. Also, the first weeks postpartum and the rapid adaptation in the physiological status are important aspects to explore further.

In the linear regression model, we included and adjusted for a given number of relevant confounders as age, pre-pregnancy BMI, parity, gestation week, P-cotinine and alcohol intake. Self-reported smoking status and current smoking by measurement of the P-cotinine level support each other, however the number of pack years was missing and might be included in the future lifestyle questionnaire. Furthermore, smoking may affect some haematological markers. A previous study has shown that chronic-smokers have increased leukocyte count and haematocrit [37]. The Greenlandic population, being approximately 56.000 inhabitants, has a high smoking rate [16], and 46% of the participants in the present study were smokers. Hence, the adjustment for the P-cotinine level in the statistical analysis is reasonable and necessary.

Greenlandic traditional food is rich in n-3 polyunsaturated fatty acids, which is known to modulate the immune system. So, we further adjusted the model for the n-3/n-6 ratio, a biomarker of marine food intake. However, the significant associations between certain haematological markers and POPs, especially PFASs, still exist after fatty acid adjustment.

This suggests that the POP exposure can influence the haematological markers of pregnant women.

The strength of the present ACCEPT sub-study is being, mainly, the very first study exploring the association of POP exposure and haematological markers in pregnant women, secondly, being geographical representative for pregnant women in Greenland, since the total study population reflects the general small population in Greenland [18]. This specific sub-study for POPs and haematological markers was not directly designed for the purpose of the larger ACCEPT study, however we found it as a strength to explore as many of the included haematological markers as possible, e.g. the inclusion of HbA1c, URIC, TSH and TT4/TT3.

Moreover, information on several potential confounders (e.g. age, pre-pregnancy BMI, parity, gestation week, P-cotinine level, alcohol intake and fatty acids) was available and taken into account in the analyses.

The limitation of our study is the low number of study participants and thus low statistically power, which may give selection bias. In addition, the convenient sampling method is critical, since the specimen in Nuuk was handled immediately, compared to the specimen included in remote districts. This may give discrepancy in the analysis of samples. The “collection site” was investigated as a potential confounder by the principle “change in estimates” – however, it was not identified as a confounder. Therefore, the storage methods seems to be acceptable and being the conditions in Greenland, due to the sparse infrastructure and centralised laboratory in Nuuk. In relation to baseline information, the study is missing adequate information regarding recent or previous infections, and diseases in pregnancy, which could have been a potential confounder. Also, this study measures the immune response through haematological markers as indirect indicators of the mother’s immune system. It could have been ideal to investigate the immune response directly e.g. through well-established marker low-grade inflammation, such as T-cells, interleukins or via high-sensitivity C-reactive protein (hsCRP) in association with serum POP levels. Furthermore, the inclusion in different gestation weeks is critical in relation to the serum POP levels, since the pregnant women’s physiological status can affect the contaminants during pregnancy.

As presented in Supplementary Table 1, few of the haematological markers showed a significant difference in relation to the trimester groups. These findings suggest a physiological change of haematological markers during pregnancy. Preferably, the present study should have included blood samples drawn in each trimester and postpartum for each participant, however, due to the economic constraints and centralised hospital in Greenland, this was not an option. However, it would be a relevant method to include in future investigations. Nevertheless, we did not observe significant difference of POPs between trimester groups, suggesting the relatively constant POP levels during pregnancy for the pregnant women in the present population.

## Conclusion

In summary, we found inverse associations between POP variables and several haematological markers in Greenlandic pregnant women. Especially in relation to blood cell counts, suggesting immunosuppressive potential of POPs. The results in the present study may be useful for assessment in clinical decisions in Inuit pregnant women. The effects of exposure to POPs on haematological markers during pregnancy need further investigation, especially in relation to immune responses in the early childhood.
