# Supplementary material for: Persistent organic pollutants and haematological markers in Greenlandic pregnant women: the ACCEPT sub-study
Source: Int J Circumpolar Health. 2018 Mar 29;77(1):1456303. doi: 10.1080/22423982.2018.1456303 (PMC5912198; doi:10.1080/22423982.2018.1456303)
Supplement: Supplementary_material.docx [file ZICH_A_1456303_SM7786.docx]

Persistent organic pollutants and hematological markers in Greenlandic pregnant women:

The ACCEPT sub-study

**Supplementary table 1, supplementary table 2 and supplementary material**

| **Supplementary table 1**. Specific hematological markers for the trimester groups (N = 189) | | | | | | | | |
| --- | --- | --- | --- | --- | --- | --- | --- | --- |
| **Parameter** | **Abbreviation** | **SI unit** | **General reference range** | **1^st^ & 2^nd^ trimester** (< 28 weeks) | | **3^rd^ trimester** (> 29 weeks) | | *p-value*^a^ |
|  |  |  |  | n | n = 114  Median (Min – Max)  *2.5^th^ – 97.5^th^ percentile* | n | n = 75  Median (Min – Max)  *2.5^th^ – 97.5^th^ percentile* |  |
| Hemoglobin | Hgb | mmol/L | 7.0 – 9.4^1^ | 110 | 7.2 (5.6 – 8.8)  *5.8 – 8.4* | 58 | 7.2 (5.6 – 8.7)  *5.6 – 8.6* | 0.494 |
| Hematocrit | Hct | ratio ^6^ | 0.35 – 0.47^1^ | 110 | 0.38 (0.27 – 0.49)  *0.32 – 0.47* | 58 | 0.40 (0.29 – 0.47)  *0.30 – 0.47* | 0.078 |
| Glycated hemoglobin A1c | HbA1c | mmol/mol | 31 – 44^1^ | 88 | 24.4 (13.8 – 44.6)  *15.7 – 32.6* | 60 | 26.5 (17.7 – 38.4)  *17.8 – 37.0* | **0.001** |
| Basophil count | BASO | 10^9^/L | 0.0 – 0.20^2^ | 110 | 0.02 (0.01 – 0.15)  *0.01 – 0.08* | 58 | 0.02 (0.01 – 0.14)  *0.01 – 0.13* | 0.475 |
| Eosinophil count | EO | 10^9^/L | 0.0 – 0.50^2^ | 108 | 0.10 (0.02 – 0.45)  *0.03 – 0.38* | 54 | 0.09 (0.00 – 0.81)  *0.01 – 0.69* | 0.776 |
| Lymphocyte count | LYMPH | 10^9^/L | 0.70 – 4.80^2^ | 108 | 1.58 (0.26 – 3.14)  *0.71 – 2.56* | 52 | 1.48 (0.80 – 2.37)  *0.80 – 2.36* | 0.292 |
| Monocyte count | MONO | 10^9^/L | 0.0 – 1.10^2^ | 108 | 0.37 (0.02 – 1.22)  *0.07 – 0.95* | 54 | 0.43 (0.14 – 0.79)  *0.16 – 0.79* | 0.073 |
| Neutrophil count | NEU | 10^9^/L | 1.80 – 7.40^2^ | 108 | 5.36 (0.86 – 11.70)  *1.04 – 10.01* | 52 | 5.45 (0.71 – 11.52)  *1.05 – 11.28* | 0.227 |
| Mean corpuscular hemoglobin | MCH | fmol/L | 1.7 – 2.1^1^ | 110 | 1.9 (1.4 – 2.1)  *1.5 – 2.1* | 58 | 1.9 (1.5 – 2.0)  *1.6 – 2.0* | 0.391 |
| Mean corpuscular hemoglobin concentration | MCHC | mmol/L | 18.6 – 22.3^1^ | 110 | 18.7 (15 .8 – 22.3)  *16.5 – 21.4* | 58 | 18.0 (15.8 – 21.5)  *15.9 – 21.2* | **0.003** |
| Mean erythrocyte corpuscular volume | MCV | fL | 81 - 109^1^ | 110 | 98 (79 – 118)  *84 - 116* | 58 | 103 (78 – 119)  *80 - 119* | **0.035** |
| Mean platelet volume | MPV | fL | 6.4 – 11.00^3^ | 110 | 10.2 (8.0 - 13.3)  *8.4 – 12.2* | 58 | 10.5 (7.4 – 12.8)  *7.9 – 12.8* | 0.114 |
| Platelet distribution width | PDW | fL | 8.9 – 15.5^4^ | 110 | 11.7 (8.0 – 18.5)  *8.7 – 17.3* | 58 | 12.4 (7.4 – 17.9)  *8.2 – 17.9* | **0.053**^#^ |
| Plateletcrit | PCT | ratio ^7^ | 0.13 – 0.34^4^ | 110 | 0.027 (0.016 – 0.066)  *0.017 – 0.046* | 58 | 0.027 (0.009 – 0.045)  *0.013 – 0.044* | 0.642 |
| Platelet count (thrombocyte count) | PLT | 10^9^/L | 135 – 400^1^ | 110 | 258 (134 – 653)  *151 - 534* | 58 | 260 (85 – 490)  *115 - 482* | 0.788 |
| Platelet – larger cell ratio | P-LCR | ratio ^8^ | - | 110 | 0.266 (0.106 – 0.497)  *0.127 – 0.421* | 58 | 0.285 (0.070 – 0.456)  *0.100 – 0.448* | 0.134 |
| Red blood cell count | RBC | 10^12^/L | 3.66 – 5.10^1^ | 110 | 3.93 (2.99 – 5.20)  *3.14 – 4.67* | 58 | 3.86 (2.89 – 4.76)  *2.94 – 4.66* | 0.890 |
| Red cell distribution width (standard deviation) | RDW-SD | fL | 39.0 – 46.0^5^ | 110 | 48.4 (38.7 – 98.3)  *40.2 – 71.0* | 58 | 53.4 (40.5 – 67.7)  *40.6 – 66.6* | **0.027** |
| Red cell distribution width (cell volume) | RDW-CV | ratio ^9^ | 0.116 –0.146^5^ | 110 | 13.9 (11.8 – 26.2)  *12.1 – 19.4* | 58 | 14.6 (12.3 – 19.3)  *12.5 – 19.1* | 0.153 |
| White blood cell count (leukocyte count) | WBC | 10^9^/L | 3.0 – 8.5^1^ | 110 | 7.5 (1.6 – 14.9)  *2.1 – 13.1* | 58 | 7.6 (2.1 – 15.1)  *2.6 – 14.0* | 0.789 |
| Thyroid stimulating hormone | TSH | mIU/L | 0.4 – 3.8^1^ | 88 | 0.8 (0.01 – 4.4)  *0.1 – 2.7* | 44 | 0.9 (0.1 – 2.5)  *0.1 – 2.4* | 0.236 |
| Thyroxine (total) | TT_4_ | nmol/L | 60 – 140^1^ | 87 | 120 (81 – 240)  *81 – 186* | 44 | 119 (86 – 176)  *86 – 176* | 0.473 |
| Triiodothyronine (total) | TT_3_ | nmol/L | 1.10 – 2.38^1^ | 87 | 2.50 (1.65 – 4.11)  *1.85 – 3.99* | 43 | 2.29 (1.54 – 3.23)  *1.55 – 3.21* | **0.001** |
| Uric acid | URIC | mmol/L | 0.15 – 0.35^1^ | 110 | 0.23 (0.11 – 0.40)  *0.14 – 0.33* | 59 | 0.23 (0.12 – 0.40)  *0.14 – 0.38* | 0.180 |
| N: total number of participants in the study population. n: number of participants with data to the corresponding parameter*.* Missing data: N – n.  ^a^ *p-value* calculated with independent t-test. ^#^ Borderline significant *p-value.* General reference range given as: ^1^ local recommendation for the Central Laboratory at Queen Ingrid Hospital, Greenland, ^2^ [22], ^3^ [23], ^4^ [24], ^5^ [25], - = no given reference range. ^6^ Hct ratio: volume fraction of MCV and RBC in %. ^7^ PCT ratio: platelet count in the blood as a percentage to the whole blood volume. ^8^ P-LCR ratio: the percentage of the platelets with a size of more than 12 fL. ^9^ RDW-CV ratio: RDW-SD / MCV. | | | | | | | | |

| **Supplementary table 2**. The n-3/n-6 ratio, total lipid and POP levels for the trimester groups (N = 189) | | | | | | |
| --- | --- | --- | --- | --- | --- | --- |
| **Parameter** | **Unit** | **1^st^ & 2^nd^ trimester** (< 28 weeks) | | **3^rd^ trimester** (> 29 weeks) | | *p-value*^a^ |
|  |  | n | n = 114  Median (Min – Max) | n | n = 75  Median (Min – Max) |  |
| n-3/n-6 ^1^ | ratio | 114 | 0.23 (0.09 - 0.87) | 75 | 0.20 (0.08 - 0.55) | 0.02 |
| Total lipid ^2^ | g/L | 107 | 8.50 (5.70 - 14.0) | 69 | 9.60 (7.10 - 14.0) | **<0.0001** |
| Level of POPs ^3^ |  |  |  |  |  |  |
| sumPCBs ^4^ | µg/kg lipid | 107 | 188.8 (57.1 - 2714) | 69 | 189.5 (55.7 - 1129) | 0.70 |
| sumOCPs ^5^ | µg/kg lipid | 107 | 241.8 (41.1 - 2198) | 69 | 239.3 (41.8 - 1985) | 0.74 |
| sumPFASs ^6^ | ng/mL | 113 | 18.7 (6.14 - 95.2) | 74 | 17.66 (6.76 - 96.4) | 0.97 |
| N: total number of participants in the study population. n: number of participants with data to the corresponding parameter*.* Missing data: N – n.  ^a^ *p-value* calculated with independent t-test.^1^ n-3: omega-3 polyunsaturated fatty acids, n-6: omega-6 polyunsaturated fatty acids. ^2^ Total lipid = 1.677 (TC-FC)+FC+TG+PL, TC: total cholesterol, FC: free cholesterol, TG: triglycerides and PL: phospholipids. ^3^ POPs: persistent organic pollutants. ^4^ sumPCBs: sum of 14 polychlorinated biphenyls. ^5^ sumOCPs: sum of 11 organochlorine pesticides. ^6^ sumPFASs: sum of 15 perfluoroalkylated substances. For single POP congeners and sum of n-3 and n-6 fatty acids, see reference [19]. | | | | | | |

**Supplementary material**

Supplementary table 1 presents the hematological markers in relation to the general reference range and trimester groups. Data shows the median, 2,5th – 97,5th percentile and minimum-maximum. Pregnant women in 1st trimester (gestation week < 12) counted only 5 participants, thus 1st and 2nd trimester were merged. Few of the hematological markers were significantly different between the two trimester groups (1st & 2nd trimester vs. 3rd trimester). HbA1c, MCV, and RDW-SD had a significantly lower median value, while MCHC and TT3 had a significantly higher median value for women in 1st & 2nd trimester, than women in 3rd trimester. A trend to higher median PDW in 3rd trimester was found. These few findings indicate a physiological change of hematological markers during pregnancy, which is further discussed in the manuscript. The results need additional investigations.

Supplementary table 2 shows the n-3/n-6 ratio, total lipid and levels of POPs in relation to the trimester groups (1st & 2nd trimester vs. 3rd trimester). Pregnant women in 3rd trimester had a significantly higher level of total lipid, compared to women in the 1st & 2nd trimester group, which suggest a change in the lipid profile towards the end of the pregnancy. Neither the lipid-adjusted PCB and OCP levels nor PFAS levels differ between trimester groups.
